# The prevalence of causes of cell mediated food allergy in children in Rio de Janeiro

**DOI:** 10.1186/1939-4551-8-S1-A282

**Published:** 2015-04-08

**Authors:** Isaac Azevedo Tenorio, Aderbal Sabra, Selma Sabra

**Affiliations:** 1 Unigranrio, Brazil

## Introduction

The frequency of Food Allergy (FA) has been increasing in recent years. The prevalence of cell-mediated FA among this cases is unknown. The major cause for the difficulty of this diagnosis of cell-mediated FA is the absense of a major biomarquer. In this study we select patients with cell-mediated FA based in their Clinical Picture, normal IgE and clinical cure with the treatment with the withdrow of the offending food from the diet.

## Material and methods

71 patients were selected for the study, with cell-mediated FA. In this group of patients we caracterise their clinical picture.

## Results

The clinical diagnosis among all the 71 patients studied was: in 62% (44 patients) the clinical diagnosis was Cow’s Milk Enteropathy (CME); in 15.5% (11 patients) the clinical diagnosis was Breast Milk Colitis (BMC); in 8.45% (6 patients) the clinical diagnosis was Abdominal Pain and Abdominal Distention with Motor Impairment (APAD); in only 5.6% (about 4 patients) the clinical diagnosis was Cow’s Milk Enterocolitis( FPIES); in others 5.6% (4 patients) the clical diagnosis was Constipation (C) and finaly in 2.8% (in 2 patients) the clinical diagnosis was Celiac Disease.

## Conclusion

The highest prevalence among all cases of FA cell ltype mediated was CME, the second clinical picture in frequency was BMC. Less frequent causes of cell mediated FA in Rio de Janeiro was APAD, FPIES, C and CD.

